# Molecular imaging of breast cancer: present and future directions

**DOI:** 10.3389/fchem.2014.00112

**Published:** 2014-12-18

**Authors:** David Alcantara, Manuel Pernia Leal, Irene García-Bocanegra, Maria L. García-Martín

**Affiliations:** Laboratory of Metabolomics and Molecular Imaging, BIONAND, Centro Andaluz de Nanomedicina y Biotecnología (Junta de Andalucía, Universidad de Málaga)Malaga, Spain

**Keywords:** breast cancer, molecular imaging of breast, breast cancer diagnosis, contrast agents, breast imaging techniques, breast magnetic resonance imaging

## Abstract

Medical imaging technologies have undergone explosive growth over the past few decades and now play a central role in clinical oncology. But the truly transformative power of imaging in the clinical management of cancer patients lies ahead. Today, imaging is at a crossroads, with molecularly targeted imaging agents expected to broadly expand the capabilities of conventional anatomical imaging methods. Molecular imaging will allow clinicians to not only see where a tumor is located in the body, but also to visualize the expression and activity of specific molecules (e.g., proteases and protein kinases) and biological processes (e.g., apoptosis, angiogenesis, and metastasis) that influence tumor behavior and/or response to therapy. Breast cancer, the most common cancer among women and a research area where our group is actively involved, is a very heterogeneous disease with diverse patterns of development and response to treatment. Hence, molecular imaging is expected to have a major impact on this type of cancer, leading to important improvements in diagnosis, individualized treatment, and drug development, as well as our understanding of how breast cancer arises.

## Introduction

Modern clinical cancer treatments require precise positional information. Where is the tumor located? How large is it? Is it confined, or has it spread to the lymph nodes? Does it involve any critical anatomical structures that would alter the treatment strategy? These questions are being answered, at ever-increasing spatial resolution, through the application of traditional anatomical imaging methods such as computed x-ray tomography (CT), magnetic resonance imaging (MRI), and ultrasound (US). Although these methods still represent the mainstay of clinical imaging, it has become clear that the acquisition of molecular and physiological information by nuclear magnetic resonance and optical imaging technologies could vastly enhance our ability to fight cancer (Weissleder, 2006).

Emerging genomic and proteomic technologies have the potential to transform the way in which breast cancer is clinically managed. Molecular imaging is poised to play a central role in this transformation, because it will allow the integration of molecular and physiological information specific to each patient with anatomical information obtained by conventional imaging methods. The hope is that clinical molecular imaging will one day be used to achieve the following: (i) the early detection of molecular or physiological alterations that signal the presence of cancer when it is still at a curable stage, (ii) the ability to evaluate and adjust treatment protocols in real time, and (iii) the ability to streamline the cancer drug development process.

The development of new breast cancer therapeutics is expensive, time-consuming, and often requires vast numbers of patients. Molecular imaging is currently one of the most powerful non-invasive techniques used in clinical diagnosis that exhibits a high potential to improve the efficiency and cost-effectiveness of drug development programs. In this article we present a short review on the main techniques and the perspectives of future Breast Cancer Imaging.

## Imaging techniques for breast cancer

Mammography and ultrasound are the most common methods used for diagnosis and guided intervention in breast disease. The relevance of breast MRI has been also increased, fulfiling an important role in operated breasts and suspicious lesions. Multiple diagnostic techniques, including tomosynthesis, mammography and ultrasound contrast elastography, 3D ultrasound, diffusion and perfusion and breast spectroscopy, have also been developed. Moreover, the use of the American College of Radiology (ARC) BIRADS scale (Breast Imaging Reporting and Data System) has been implemented in diagnostic centers during the last decade (American College of Radiology, 2003). BIRADS classification started in the late 1980s to address a lack of standardization and uniformity in mammography practice reporting (McLelland et al., 1991). The BIRADS lexicon provided new opportunities for quality assurance, communication, research, and improved patient care. Many well-respected groups participated in this development initiative to establish a broad base of support (D'orsi and Kopans, 1997; Burnside et al., 2009; Mercado, 2014).

**Figure 1:**
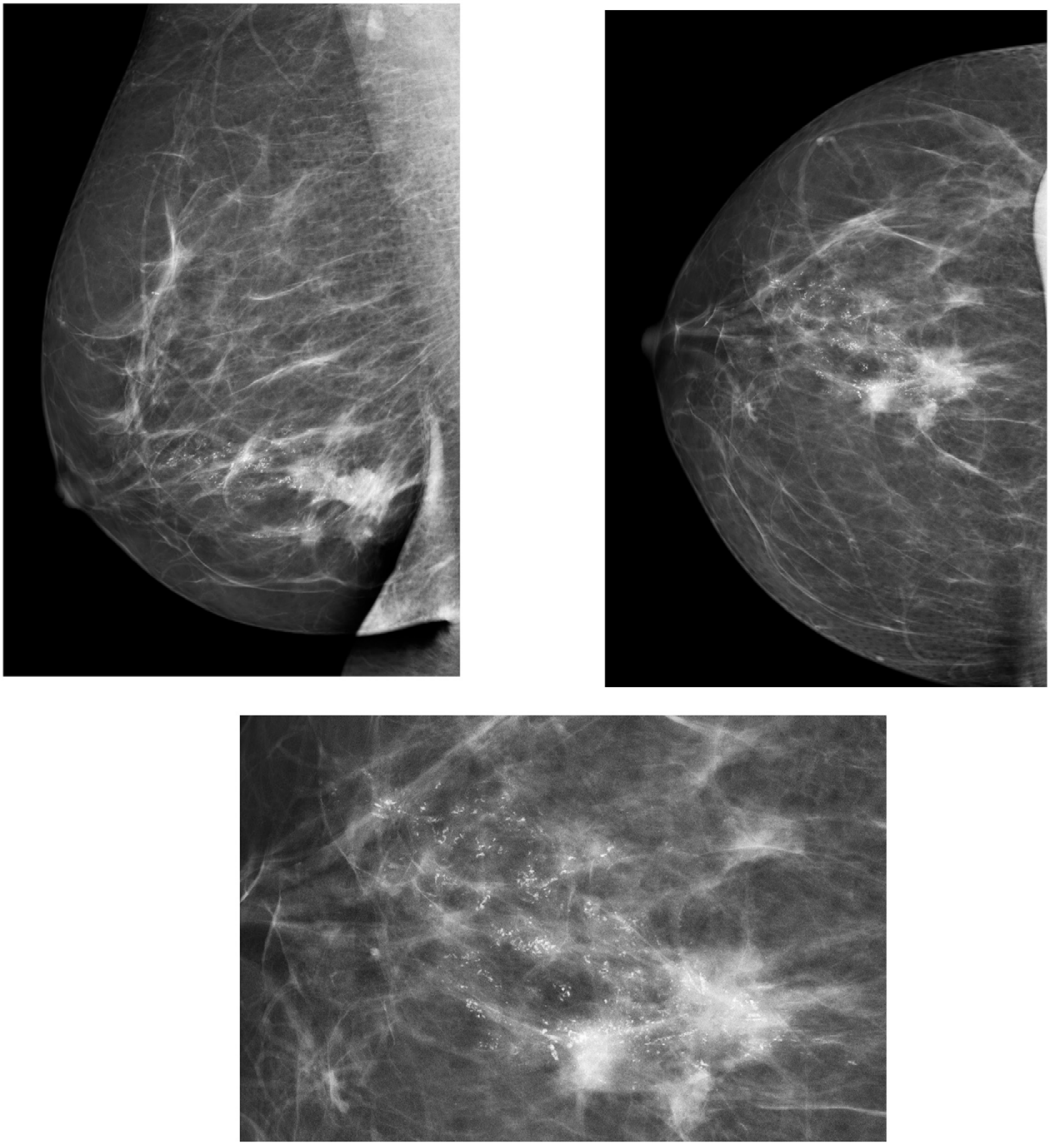
**Microcalcifications in breast mammography.** Highly suggestive of malignancy BIRADS 5.

The BIRADS scale is a classification of breast disease according to radiological findings that includes six grades of malignancy and indicates the actions that must be followed for each grade (Figure 1). The implementation of BIRADS has allowed us to homogenize the diagnosis and injury treatment methods in all centers.

### BIRADS classification

**Table:** 

**Category 0:**	Additional imaging evaluation and/or comparison to prior mammograms is needed.
**Category 1:**	Negative.
**Category 2:**	Benign (non-cancerous) finding.
**Category 3:**	Probably benign finding – Follow-up in a short time frame is suggested.
**Category 4:**	Suspicious abnormality – Biopsy should be considered:
	**Category 4A:** Finding with a low suspicion of being cancer.
	**Category 4B:** Finding with an intermediate suspicion of being cancer.
	**Category 4C:** Finding of moderate concern of being cancer, but not as high as Category 5.
**Category 5:**	Highly suggestive of malignancy – Appropriate action should be taken.
**Category 6:**	Known biopsy-proven malignancy – Appropriate action should be taken.

The main diagnosis and monitoring techniques of breast disease include mammography, ultrasound and magnetic resonance imaging (MRI).

### Mammography

Mammography is the most commonly used method for the monitoring and diagnosis of breast disease. In recent years, digital mammography has been developed, which requires lower doses of radiation as compared to a conventional mammography, and also allows the post-processing of images.

Mammography is also used to guide interventional breast techniques, such as stereotactic mammography. Two mammographic views, cranio-caudal and oblique medium lateral are usually performed and complemented with other projections according to needs. This technique has high sensitivity for the diagnosis of microcalcifications. It is also used for breast screening, allowing the detection of breast lesions at a very early stage, which increases considerably the life expectancy of affected patients. According to the screening program, a mammography is usually performed every two years (two projections) in women over the age of 40 or 50, with double read by two different radiologists (Houssami et al., 2009).

However, mammography still present some drawbacks. Firstly, it is an ionizing technique, and although the radiation dose has considerably decreased, it is still relevant if we take into account that the breast is a radiosensitive tissue. Secondly, mammography cannot differentiate between liquid lesions, including cysts, and solid lesions, which is a major limitation for the accurate identification of tumor masses.

Two innovative techniques are included within mammography:

#### Contrast-enhanced mammography

Contrast mammography, as well as MRI, allows dynamic vascular studies to be performed. Several parameters can be extracted from the enhancement curve that provide useful diagnostic information, such as the slope and the time to peak. Thus, lesions with early and intense enhancement are suggestive of malignancy (Fallenberg et al., 2014).

This type of study has been satisfactorily used for the analysis of inconclusive lesions, the detection of occult lesions, the monitoring of disease progression and to assess chemotherapy response (Dromain et al., 2009).

#### Tomosynthesis

Tomosynthesis techniques allow us to carry out three-dimensional breast studies. It consists of a mammography device that uses a rotary head tube, performing different projections of a static breast with a specified angle (between 15° and 45°). It can be considered a tomographic application of digital mammography. Tomosynthesis has even been proposed as a new screening method (Waldherr et al., 2013). Tomosynthesis has demonstrated superior accuracy compared to mammography in tumor measurements and reduced the suspicious presentations of normal tissues and tissue overlap, and facilitated accurate differentiation of lesion types (Fornvik et al., 2010; Alakhras et al., 2013).

### Ultrasound

**Figure 2:**
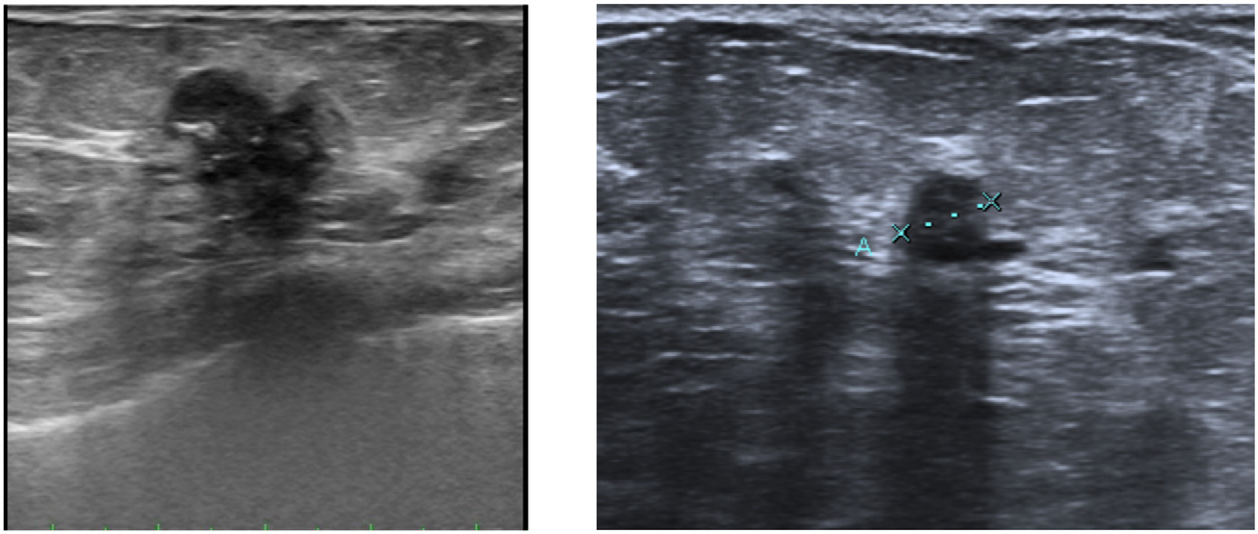
**Breast cancer ultrasound images**.

Ultrasound complements mammography, being a required method for the management and diagnosis of breast pathology (Figure 2). Because it does not use ionizing radiation, ultrasound is not only the first diagnostic tool in young women who have little risk of breast cancer, but also the first diagnostic technique in pregnant and breastfeeding women. Moreover, it has high sensitivity to show tumor margins and the internal characteristics of tissue, and it is used as complementary technique to mammography for the study of dense breasts and to assess lymph node status. It is also frequently used for breast intervention, for guided biopsy and for the placement of harpoons. In recent years, different ultrasound techniques for the breast pathology studies have been developed, including:

#### Ultrasound contrast

Ultrasound contrast involves the intravenous injection of perfluorocarbon microbubbles to observe the behavior of breast lesions by ultrasound. The perfusion area and the signal intensity curves in relation to time are obtained using this method. Ultrasound contrast is used for diagnosis, detection of recurrence and monitoring of treatment response, and is particularly useful as a guide for puncture of suspicious lymph nodes, as metastatic lymph node areas do not capture contrast and can be therefore differentiated from healthy areas.

On the other hand, 3D ultrasound is particularly useful for the study of breast lesion with contrast, because it allows the assessment of nodes with contrast in three dimensions (Jia et al., 2014).

#### Elastography

This technique is based on the same principle as breast tenderness and can determine the hardness of the lesion by measuring the elastic properties of tissues by ultrasound, as the lower the hardness of a lesion, the higher the probability of being benign, and vice versa. Elastography has been shown to be very useful in the assessment of benign lesions (BIRADS 3) (Itoh et al., 2006; Scaperrotta et al., 2008).

### Magnetic resonance imaging (MRI)

MRI is an important diagnostic tool frequently used to study breast disease. It currently has specific indications, including evaluation of response to treatment, screening in high-risk patients, study of occult breast cancer, study of tumor recurrence and assessment of breast prostheses. MRI can be also recommended for the staging of breast cancer, the study of microcalcifications, breast discharge, premalignant lesions, residual tumor in operated patients or in case of inconclusive findings by mammography and ultrasound (Mann et al., 2008; Sardanelli et al., 2010).

The MRI techniques applied to the study of breast cancer are based on both the assessment of the morphological features of the lesions and the characteristics of contrast enhancement of these lesions. Malignant tumors have a disorganized angiogenesis showing specific morphological and functional characteristics.

The MRI study includes pre-contrast T2 sequences and post-contrast 3D T1-weighted gradient Eco. Several parameters are analyzed in the contrast studies, such as the slope of the enhancement curve during both the uptake and wash out phases, the time to peak enhancement or the maximal relative enhancement. These analyses can differentiate benign from malignant lesions. Image post-processing plays an important role in dynamic contrast MRI because it provides the radiologist with additional parametric information that can be crucial for a more accurate diagnosis. Thus, typical post-processing processes include subtraction of images, projections of maximum signal intensity (MIP), multiplanar reconstructions (MPR) and time curves of suspicious lesions uptake (Kuhl, 2007; Partridge, 2008).

Currently, there are two classification systems for the diagnostic criteria in breast MRI: the Fischer and the ACR classifications. Both present common diagnostic criteria, integrating morphological and dynamic uptake information. The ACR classification presents common criteria with the BIRADS classification of mammography and ultrasound (Agrawal et al., 2009; Morris Ea et al., 2013). During the last few years, other MR techniques have been proposed for the study of breast cancer, namely diffusion MRI and spectroscopy.

#### Diffusion MRI

Diffusion MRI techniques are based on the application of field gradients to enhance the signal lost due to the Brownian motion of water molecules. The diffusion weighting is determined by the strength and duration of the diffusion gradients, and the time between the gradient pulses, which is all quantified by the b-factor. The exponential fit of signal intensity vs. the b values provides the apparent diffusion coefficient (ADC), whose values reflect the restriction of water motion in any given tissue and, in the case of tumor growth, it has been related to cellularity. Malignant tumors usually have high cellularity and therefore present low ADC values as compared to benign lesions. Diffusion MRI is a quick technique that does not require the use of contrast agents and its implementation has been recommended as part of a routine protocol for breast MRI. The main disadvantages of diffusion MRI are the low spatial resolution and the lack of specificity to differentiate between benign and malignant tumors (Guo et al., 2002; Peters et al., 2008).

#### MR spectroscopy

Breast spectroscopy provides information about the metabolic profile of tumor tissue, being the most important metabolite in breast spectroscopy tCho (total choline), which has been related to tumor proliferation activity. The use of spectroscopy has been shown to increase the specificity of MR for the differentiation of benign and malignant lesions. Several studies indicate the association between the choline peak with the response to the treatment. In this sense, Tozaki et al. (2010) found that the reduction of choline peak is more sensitive to determining the response to treatment than the decrease of tumor size. Furthermore, the combination of spectroscopy and diffusion MRI data have demonstrated a high specificity to characterize benign and malignant lesions (Tsougos et al., 2014).

## Contrast agents

In spite of the multiple image diagnostic tools commented on above, radiologists still find some difficulties when diagnosing early stage breast tumor malignancies due to the lack of sensibility given for these techniques. Early detection has become one of the most important issues in the cancer treatment, and researchers have improved this issue by developing external substances called contrast agents that enhance the sensibility of images in the region of interest. This fact allows improvement to the quality and the follow-up of molecular processes at the cellular and molecular levels of the region under study. The more common contrast agents used in clinical studies are gadolinium- and iodine-based structures for magnetic resonance imaging and mammography (X-ray) respectively, however other contrast agents such as radiotracers are being investigated as potential biomarkers for daily clinical practice.

### Magnetic resonance imaging contrast agents

As noted above, the gadolinium-based contrast agents are the most commonly used in clinical practice, generating a positive image of the nearby tissues. The presence of gadolinium ions shortens the T_1_ relaxation time, thus generating an increase of the intensity in the images (bright signal or positive image) that help to distinguish malignancies from other benign pathologies (Zhou and Lu, 2013). However, gadolinium is a high toxic paramagnetic cation (Gd^3+^) that needs to be protected from the body, so gadolinium is mainly reacted with chelate ligands thus minimizing the toxicity effects from the free gadolinium (Gd^3+^). Therefore, the most typically used gadolinium chelates as MRI contrast agents in clinical practice are gadopentetate dimeglumine (Gd-DTPA, Magnevist®), gadoterate dimeglumine (Gd-DOTA, Dotarem®), gadoteridol (Gd(HP-DO3A), Prohance®), and gadodiamine (Gd(DTPA-BMA), Omniscan®). These contrast agents, approved by the Food and Drug Administration (FDA), present excellent biodistribution within the extracellular space, and fast renal clearance from the body with half-lives of 1–2 h. Albeit the most used gadolinium-based contrast agents are the Gd-chelates, complex gadolinium-based contrast agents have been developed to improve their T1 relaxation time and pharmacokinetics. These complexes provide well-defined advantages over Gd-chelates due to slow rotational motion, they are a combination of Gd-chelates, such as Gd-DTPA and Gd-DOTA, and dendrimers (Li et al., 2013) or liposomes (Huang and Tsourkas, 2013; Zhou and Lu, 2013). For instance, poly(amidoamine) PAMAM dendrimers in different generations have shown higher r1 relaxivity and size-dependent pharmacokinetics, low generation (2–4) presented renal clearance, and high generation (5–10) presented minimal renal clearance. A recent modification in the design of contrast agent dendrimers resulted in the development of dendrimer nanoclusters (DNCs), as a combination of small PAMAM dendrimers. These DNCs were easily synthesized in high yields and also exhibited higher r1 values than the small units of PAMAM-based contrast agents (Cheng et al., 2010). These Gd-chelates were also combined with liposomes via encapsulation in the inner core or immobilization at the liposome surface, being the immobilization most used since the relaxivity is higher than in the encapsulated method, thus exhibiting a low water exchange rate with the gadolinium encapsulated in the inner core of the liposome.

### X-ray contrast agents

Iodine-based contrast agents improve the visualization of images in radiography and CT by increasing the density of tissues and also the vessels. Similarly to dynamic MRI using Gd chelates, relevant information can be extracted from the different uptake kinetics of these contrast agents that helps in differentiating malignant lesions from benign tissues. In case of malignant tissues, the iodine is rapidly absorbed and desorbed (more contrast), while in the benign ones the absorption takes place slowly (less contrast), thus giving differences in the tissue density of the images. Iodine contrast agents could be sorted into two groups depending on the binding to the iodine: covalent (non-ionic) or ionic contrast agents (Robbins and Pozniak, 2010). The ionic contrast agents present are better contrast agents than the non-ionic ones due to their higher osmolality, with the consequently increased delivery or disassociation of iodine (Barrett et al., 1992). However, the ionic iodine contrast agents induces more toxicity (more side effects) mainly due to the large amount of iodine ions delivered (higher osmolality injected in comparison with serum), being also recently reported that these contrast agents could affect the thyroid in some patients (Rhee et al., 2012). Therefore, in order to minimize the toxicity, the contrast agents used for the clinical practice present values close to the serum, and non-ionic bonds such as Iohexol 300 mg Iodine/mL (Omnipaque) and Iodixanol 320 mg Iodine/mL (Visipaque).

### Positron emission mammography (PEM)

PEM is high-resolution PET scanner that provides functional imaging specifically for breast cancer detection (Kalles et al., 2013). PEM can isolate and enhance breast images with more accuracy than full-body PET scans and works much like a full-body PET scan (see Section PET and SPECT). In this technique, the contrast agents used in the evaluation of breast cancer are radiotracers (Penuelas et al., 2012), in particular radioactive labeled sugar-like molecules. These radiotracers help the diagnostic accuracy of the cancer, especially in the early stages, metastasis, and also cancer progression during the treatment. ^18^F-fluorodeoxiglucose (FDG) (Caldarella et al., 2014) is the most typical radiotracer used as a contrast agent in PEM. FDG is an analog of glucose that is accumulated, after injection, mostly in cancer tissues, since those present a faster metabolism in comparison with the normal tissues allowing a more clear vision of the suspected malignant tissues by PEM. Another radiotracer is the 3′-deoxy-3′-[^18^F]fluorothymidine (^18^FLT). This radiotracer is not still used as a routine breast cancer contrast agent in clinical practice, but it has been tested as a biomarker for imaging cellular proliferation. ^18^FLT is a structural analog of DNA nucleoside thymidine that is trapped in the cell thus informing about the stage or monitoring the evolution of the tumor cells (Caldarella et al., 2014). In addition to the imaging of breast cancer proliferation and progression, the N-[^11^C]methylcholine (^11^C-choline) is used as a radiotracer (Contractor et al., 2009) since the choline is modified to phosphocholine due to the increase of the activity of the enzyme choline kinase-α as noted above in the MR spectroscopy section.

## Molecular imaging techniques for breast cancer

The term “molecular imaging” refers to the non-invasive visualization and measurement of biological processes at the cellular and molecular levels in a living system using endogenous or exogenous markers.

There are many different imaging modalities that can be used for molecular imaging, the most relevant ones being: nuclear imaging (PET and SPECT), optical imaging and magnetic resonance imaging.

The direct observation of endogenous markers can be achieved with magnetic resonance *in vivo* spectroscopic imaging (MRSI) (Begley et al., 2012; Bolan, 2013) or some advanced optical methods, such as Raman spectroscopy (Kallaway et al., 2013). The first one is based on classical nuclear magnetic resonance (NMR) spectroscopy, which allows the detection and quantification of molecules containing magnetic nuclei, typically ^13^C, ^31^P, ^19^F or ^1^H, being ^1^H NMR the most widely used *in vivo*. The combination of NMR sequences with field gradients in MRI scanners allows for the spatial localization of the observable metabolites, giving rise to MRSI. Both ^1^H and ^31^P MRSI have been used for the metabolic characterization of breast tumors at a high magnetic field (Klomp et al., 2011). Raman spectroscopy is based on inelastic scattering of photons after interaction with vibrating molecules and thus provides information about tissue composition (Brozek-Pluska et al., 2012; Li et al., 2014).

The term molecular imaging, however, most commonly refers to the use of exogenous markers (contrast agents) to visualize and measure *in vivo* processes. For breast cancer diagnosis, PET and SPECT have been widely used in clinical practice, whereas MRI is expected to have a major impact in the near future, and optical imaging is mainly used in preclinical studies.

### PET and SPECT

PET imaging uses radioactive isotopes that emit positrons, *such as* ^18^F, ^15^O, ^13^N, or ^11^C; whereas SPECT imaging uses isotopes that emit gamma photons, such as ^99m^Tc, ^123^I, or ^125^I. Positrons travel short distances in tissues, in the order of millimeters, and collide with surrounding electrons (annihilation), producing two high energy gamma rays that travel in opposite directions to one another and are detected by the PET camera. The time delay between the detection of paired opposite direction is used to calculate the location of the annihilation event. In SPECT, a single photon is emitted per event and detected by rotating gamma cameras.

Most PET radioisotopes are short-lived, ranging from a few minutes to 2 h, which implies the availability of an on-site cyclotron to produce them and therefore increases the cost of PET imaging dramatically. SPECT radioisotopes are longer-lived, in the order of hours (6 h for ^99m^Tc), allowing for longer image acquisition times. On the other hand, PET shows higher sensitivity as compared to SPECT.

Both PET and SPECT provide information about physiological activity, such as glucose metabolism, blood flow and perfusion, and oxygen utilization (Kjaer, 2006). However, they lack anatomical detail, which has led to the development of hybrid systems that combine PET and SPECT with other image modalities, CT and MRI.

Both whole-body and dedicated PET/CT scanners are currently available. Dedicated systems have higher sensitivity allowing for the detection of small tumors and thus being more accurate for molecular imaging, whereas whole-body scanners provide valuable information for locoregional and distant staging (Koolen et al., 2012). PET/MRI is a more recent technology that offers the advantage of lower exposure to radiation and higher contrast resolution, together with the possibility of adding functional information from other MRI modalities, which has great potential for molecular imaging. However, further technological developments are still needed to get optimal performance of a fully integrated PET/MRI system (Pace et al., 2014). SPECT/CT has shown to be a valuable tool for sentinel lymph node detection (Husarik and Steinert, 2007; Lerman et al., 2007; Van Der Ploeg et al., 2009; Coffey and Hill, 2010).

### Optical imaging

Optical molecular imaging of the breast is based on the use of near-infrared (NIR) light to excite exogenous fluorescent probes that have been designed to selectively target breast tumor cells (Levi et al., 2007; Poellinger, 2012). The use of NIR-fluorophores for immunohistochemical characterization of excised tumor specimens is a common *in vitro* diagnostic technique. The goal of molecular imaging, however, is to detect these fluorophores *in vivo*, thus avoiding the need for biopsies. There are technical limitations, though, that need to be addressed if these methods are to be used on patients, like tissue penetration and background signal contamination. To date, the use of NIR optical imaging *in vivo* is limited to tumor xenografts in preclinical studies (Oliveira et al., 2012; Sano et al., 2012; Van De Ven et al., 2012) or intraoperative imaging for tumor margin detection and lymph node mapping (Lee et al., 2010; Verbeek et al., 2014).

### Molecular magnetic resonance imaging

**Figure 3:**
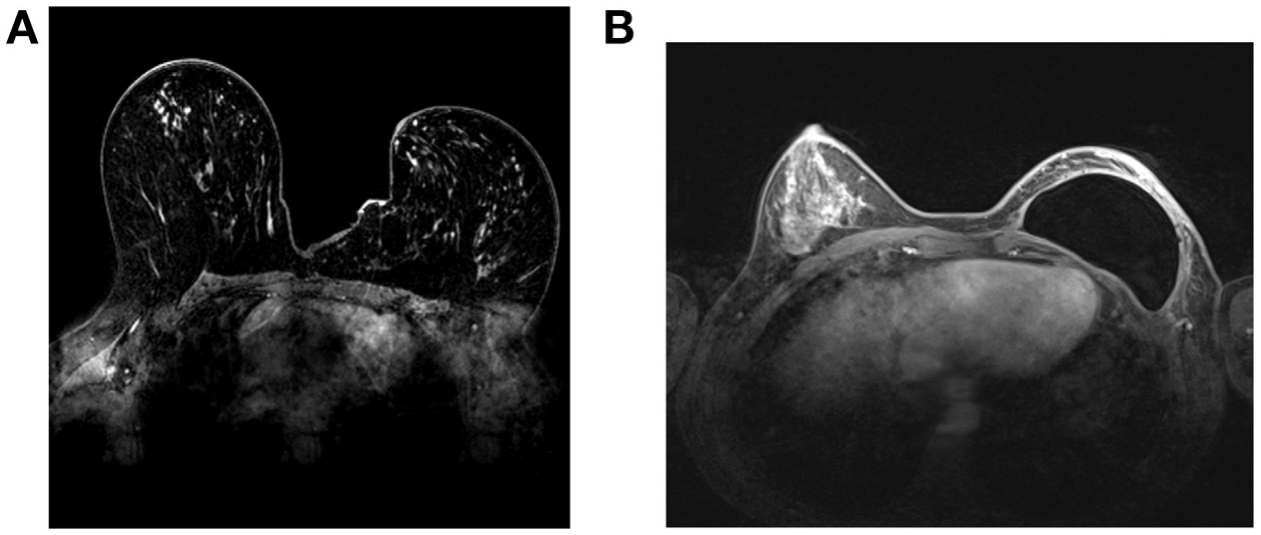
**Breast MRI images at 1.5T**. A Conventional breast MRI image (without contrast agent) **(A)**. A breast prosthesis can be seen in the right breast **(B)**.

MRI has attracted a great deal of interest in the era of molecular imaging, as it is the most versatile diagnostic imaging modality, able to provide excellent anatomical detail, together with functional and metabolic information (Figure 3). Furthermore, its non-ionizing nature offers the possibility of performing longitudinal follow-up studies without any risk for the patient.

The majority of the MRI signal comes from the water protons (^1^H) and the contrast from the local differences in water content, water motion and magnetic relaxation times, T_1_ and T_2_, of the water protons. Although intrinsic contrast is sufficient for most MRI applications, the use of exogenous contrast agents is often required for accurate diagnosis. Most MRI contrast agents are based on either gadolinium chelates or superparamagnetic nanoparticles (SPIONs), these being the base for molecular imaging.

In contrast to nuclear and optical imaging modalities, which are based on the direct detection of molecular probes, molecular magnetic resonance imaging is an indirect method that detects the effect of the contrast agents on the magnetic properties of the surrounding water molecules. This is a crucial aspect in understanding MRI-based molecular imaging, as explained below.

The major drawbacks of MRI for molecular imaging applications are, on the one hand, its inherent low sensitivity, due to the small difference in atoms between the high and the low energy states, and on the other hand, the lack of specificity of conventional MRI contrast agents. Thus, molecular imaging probes have to be able to strongly increase sensitivity and at the same time show high specificity. In this regard, SPIONs (Lodhia et al., 2010; Ittrich et al., 2013; Jin et al., 2014) have important advantages over other magnetic contrast agents because they produce signal enhancement through local field inhomogeneities, which affects the T2 of a large number of water molecules, thus leading to very strong signal enhancement. Nonetheless, other types of contrast agents have also been proposed for magnetic resonance molecular imaging, such as Gd-based nanoparticles (Huang and Tsourkas, 2013). Finally, the functionalization of these nanosystems using technological approaches adds both specificity and biocompatibility.

Some studies have already shown the great potential of magnetic resonance for molecular imaging of breast cancer using targeted nanoparticles (Li et al., 2013; Yan et al., 2013). Although these studies have been only conducted in animal models, it can be expected that, in the near future, the rapidly growing field of nanomedicine will facilitate the translation of these methodologies to the clinics.

## Perspectives

Although molecular imaging is able to visualize breast tumor morphology and functional and metabolic processes within the tumor at several levels, the sensitivity of the different molecular imaging techniques is varied depending on the type of marker used in signaling the biological processes. At present, the main milestones for future molecular imaging development in breast cancer are:

1. To enhance knowledge of molecular drivers behind breast cancer subtypes, progression and metastasis.
2. To develop validated markers for chemosensitivity and radiosensitivity.
3. To validate multimodality imaging biomarkers for minimally invasive diagnosis and monitoring of responses in primary and metastatic disease.
4. To develop interventions and support to improve the survivorship experience.

In 2012, the charity Breast Cancer Campaign facilitated a series of workshops where specialists and other stakeholders revealed the main gaps in the prevention and treatment of breast cancer (Eccles et al., 2013). Top problems in molecular imaging of breast cancer (and recent research on the field) to be highlighted are:

1. There is a need to increase the use of functional screening techniques to learn about tumor heterogeneity, identify features associated with response or resistance to treatment and accelerate the rate at which promising ones enter clinical evaluation. The “Europe Against Cancer” programme has created quality assurance guidelines used for all mammography-based screening for breast cancer. They were created to maximize results while minimizing negative effects. The Mammography Quality Standards Act (MQSA) in the United States has mandated that all mammography clinics be certified (Von Karsa and Arrossi, 2013). Resistance to chemotherapy has brought to light the issue of tumor heterogeneity. Approximately 70% of human breast tumors are ER positive and depend on estrogen for growth. The use of selective ER modulators, such as tamoxifen, in ER-expressing tumors was one of the first examples for successful targeted therapy based on the tumor's molecular classification (Swaby et al., 2007). What induces endocrine resistance in these tumors has been one of the longest standing and most intense areas of breast cancer research. The somatic evolution of tumor progression was discovered in 2012, but the results raised additional questions that could not be answered at that time (Greaves and Maley, 2012). One of the most exciting outcomes of comprehensive cancer-genome-sequencing studies is that we finally have the tools to follow clonal and subclonal evolution of tumors and see the complexity of cancers as a whole (Polyak, 2014).
2. Evaluation of emerging imaging biomarkers of primary and metastatic breast cancer. A biomarker is a crucial tool for measuring the progress of disease and the effects of treatment for better clinical outcomes in breast cancer patients. The current questions of therapeutic choices can focus now on the understanding that breast cancer is truly a collection of genetically-specific heterogeneous diseases, each demonstrating different clinical behavior and therapeutic response (De Mattos-Arruda et al., 2013). Several biomarkers have been proposed as new breast cancer targets, including MicroRNAs (mi-RNA) (Mulrane et al., 2014), proteins (Kondo, 2014), antibodies (Knowles and Wu, 2012), or glycans (Adamczyk et al., 2012). One promising direction is the detection and imaging of circulating cell-free DNA (cf-DNA). Since 2002, cf-DNA has been shown to represent a good non-invasive biomarker, as it can be isolated from human plasma, serum and other body fluids (Utting et al., 2002). It was also reported that the concentration of DNA in the bloodstream of patients with breast cancer was higher than healthy controls (Fleischhacker and Schmidt, 2007). Thus, the detection of cf-DNA provides new opportunities for management of cancer patients, adding a useful new tool for diagnosis, staging and prognosis (Esposito et al., 2014). Imaging of cf-DNA after chemotherapy treatment has been described by using fluorochrome-functionalized nanoparticles (Cho et al., 2013). Very recently, cf-DNA from plasma samples has been imaged by AFM and allowed to confirm the specific size pattern of tumor-derived cf-DNA (Mouliere et al., 2014).
3. Increased specificity and improved clinical translation of radiotracers for positron emission tomography/single-photon emission computed tomography (PET/SPECT). Since the discovery of GLUT family proteins overexpression associated with certain tumors, a variety of radiolabeled glucose derivatives have been developed as SPECT and PET tumor imaging agents. [18F]FDG is by far the most widely used in PET imaging for cancer diagnosis. Unfortunately, clinical usage is limited due to the need for the presence of cyclotron in 18F production. Generator produced isotopes, such as 99mTc and 68Ga, are readily available and affordable. The availability of a generator and kit chemistry to prepare 99mTc and 68Ga-based molecular probes may have a significant impact on nuclear medicine (Liu et al., 2014). It has been shown that 99mTc-glucarate may behave as a suitable alternative to 18F-FDG as a promising breast tumor imaging agent and needs to be further investigated (Gambini et al., 2011). Thus, using generator-produced isotopes to label glucose analogs is the major focus of ongoing research.
4. Identification and assessment of using imaging biomarkers currently associated with other cancer indicators in additional hallmarks such as hypoxia, invasion and changes in metabolism. During the past decades, researchers have tried to elucidate the mechanisms that underlie cancer-related death. However, this remains a challenge, as genomic instability causes a constantly changing genetic profile of tumors, and local variations in the microenvironment cause heterogeneity in tumor cell behavior (Polyak, 2014).
5. How to validate novel imaging biomarkers in adequately powered multi-center clinical trials. While applied molecular biology to cancer has made great advancements, the development of clinically validated biomarkers for primary breast cancer has remained an unconquerable task. Chemo-N0 (1993–1998) was the first prospective randomized multicenter trial in Node-negative breast cancer designed to prospectively evaluate the clinical utility of a biomarker. Its results established uPA/PAI-1 as a clinically useful biomarker for assessing long-term prognosis in early breast cancer and benefit from adjuvant chemotherapy in the high-risk group; it is thus well-suited for routine risk assessment in node-negative breast cancer (Harbeck et al., 2013). The Node Negative Breast Cancer Trial (NNBC), initiated by the Swedish Breast Cancer Group, was able to validate a prognostic index consisting of a proliferation factor, PR-status, and tumor size. The index may be helpful for prognostic considerations and for selection of patients in need of adjuvant therapy (Klintman et al., 2013). Although still in their infancy, circulating mi-RNAs and cf-DNA are beginning to be recognized as vital to future strategies on therapies for breast cancer (Ng et al., 2013; Esposito et al., 2014). mi-RNAs have become the rising stars for novel molecular targeting treatments because of their ability to regulate multiple genes in molecular pathways (Si et al., 2013). Very recently, a Phase 1 clinical study of MRX34, the first miRNA to advance into a human clinical trial for liver cancer, was approved (Mirna Therapeutics, 2014).
6. Methods of reporting intratumoral heterogeneity and locate the most beneficial areas for biopsies and radiotherapy. Within the plethora of imaging modalities, diffusion weighted magnetic resonance imaging (DW-MRI) has shown promise for the detection and characterization of breast cancer. Apparent diffusion coefficient (ADC) values allow quantification of the diffusion signal, and can facilitate in differentiating benign and malignant breast tumors as well as identifying early response in tumors undergoing preoperative treatment (Partridge and McDonald, 2013 and the references cited therein). On the other hand, the heterogeneous nature of cancer still presents an important challenge in cancer imaging and therapeutics (Seoane and De Mattos-Arruda, 2014). This heterogeneity also confers to the different breast cancer subtypes a specific invasional kinetic pattern, as has been recently shown by Yamaguchi et al. using MRI studies (Yamaguchi et al., 2014). Although breast MRI accuracy for assessing residual disease is good and surpasses other diagnostic techniques, overestimation and underestimation of residual disease have also been observed. This is largely because of the various treatment types and breast cancer subtypes (Lobbes et al., 2013). With some limitations and taking into consideration that many of the experiments have been performed in mice, intravital microscopy (IVM) is another technique that has proven its power to elucidate the cellular and molecular events that underlie the hallmarks of cancer. Fluorescence-guided surgical procedures have also benefited from IVM, which translates into more promising uses in the clinical setting (Ellenbroek and Van Rheenen, 2014).
7. Extension of methods that identify and define subtypes of cancerous tumors —DCIS, TNBC and luminal types—with non-invasive procedures (which may identify mixed lesions missed by homogenized or limited sample analyses) and assess heterogeneity between metastases. In recent years, there has been an explosion in the field of nanomedicine with the development of new nanoparticles for the diagnosis and treatment of cancer, and the related term “nanooncology” has been adopted by many (Thakor and Gambhir, 2013). The development of new contrast agents for MRI opens a new way for non-invasive breast cancer characterization. Special attention is made to iron oxide nanoparticles, currently one of the best options in clinic due to its lack of toxicity (Kievit and Zhang, 2011; Rosen et al., 2012). Meier et al. used magnetic nanoparticles (SPIONs) decorated with folic acid to image FR-positive human breast cancer cell lines non-invasively (Meier et al., 2010). Very recently, Sun et al. have developed SPIONs functionalized with extra domain-B of fibronectin (EDB-FN) peptide for *in vivo* imaging of breast tumor initiating cells (BTICs) by MRI (Sun et al., 2014). The development of new algorithms in contrast enhanced MRI is also enabling good discrimination of triple-negative cancers from non–triple-negative cancers, as well as between triple-negative cancers and benign fibroadenomas (Agner et al., 2014). This computer assisted diagnosis opens a new window for quick breast cancer identification and therapeutical match.
8. Combination of multidisciplinary inter-field data. It has been recommended that imaging studies (both preclinical and clinical) would need to be coregistered with linked genomic and proteomic information in order to fully understand the biological implications of the images registered (Segal et al., 2007; Lambin et al., 2012; Waterton and Pylkkanen, 2012). Currently, imaging studies are often separated from tissue collection due to a lack of appreciation of how the coordination could benefit.
9. Identification and evaluation of biomarkers with therapeutic responses. More extensive usage of orthotopic xenograft and transgenic murine models of primary and metastatic breast cancer will demand robust preclinical imaging approaches. Trials that make use of these images will experience increased accuracy for novel agents, which in turn can speed up the development of successful treatments and the early cessation of those that show no promise (publication of negative results has been recommended by many researchers Alcantara et al., 2010; Anderson et al., 2013). These preclinical trials might also lead to sequential and combination treatment regimens.

As has been listed above, there are many new and emerging molecular imaging technologies that can benefit breast cancer patients. Other molecular imaging procedures under development often combine imaging systems to form hybrid technologies that improve accuracy and allow physicians to see how cancer may be affecting other systems in the body. One of the more promising research areas is in investigational PET imaging biomarkers, such as fluorothymidine (FLT) and fluoroestrogen (FES). FLT has shown promise for the demonstration of tumor proliferation and FES for the demonstration of estrogen receptors. Other exciting area of study is radioimmunotherapy, a form of treatment that targets cancer-killing radiation directly to cancer cells (out of the scope of this review).

## Conclusions

The past 40 years have seen stunning improvements in the ability of noninvasive imaging to characterize structures and functions. These strides have come from the progressive evolution of conventional imaging techniques, with relatively little impact from imaging targeted to specific molecular moieties. Although the basic science of molecular imaging continues to make impressive strides, the regulatory and commercial landscape is limiting to these investigational imaging agents.

We anticipate that future needs will include the development of nanomaterials that are specific for immune cell subsets and can be used as imaging surrogates for nanotherapeutics. New *in vivo* imaging clinical tools for noninvasive macrophage quantification are thus ultimately expected to become relevant to predicting patients' clinical outcome, defining treatment options and monitoring responses to therapy.

### Conflict of interest statement

The authors declare that the research was conducted in the absence of any commercial or financial relationships that could be construed as a potential conflict of interest.
